# Beverage Consumption Patterns Among Navajo Children Aged 2–5 Years

**DOI:** 10.1016/j.cdnut.2024.104493

**Published:** 2024-10-26

**Authors:** Carmen V George, Brianna John, Kenneth Hecht, Christina Hecht, Letizia Trevisi, Laura Vollmer, Kerlissa Bitah, Eva Bennett, Louise Benally, Malyssa Egge, Rachel Whitman, Lavina Tsosie, Asia Soleil Yazzie, Sonya S Shin

**Affiliations:** 1 Division of Global Health Equity, Brigham and Women’s Hospital, Boston, MA, United States; 2 Department of Agriculture and Natural Resources, Nutrition Policy Institute, University of California, Oakland, CA, United States; 3 Depatment of Global Health and Social Medicine, Harvard Medical School, Boston, MA, United States; 4 Cooperative Extension, Division of Agriculture and Natural Resources, University of California, Davis, CA, United States; 5 T’iis Nazbas Community School, Teec Nos Pos, AZ, United States; 6 Community member, Navajo Nation, United States; 7 Northern Arizona University, AZ, United States; 8 Diné College, AZ, United States; 9 Department of Global Health and Social Medicine, Boston, MA, United States

**Keywords:** Navajo, children, water, hydration, Native American, rural, early child education, Indigenous, sugar-sweetened beverages

## Abstract

**Background:**

Child beverage habits contribute to long-term health outcomes, including health conditions shaped by diet and adequate hydration. However, little is known about beverage consumption patterns of young American Indian children.

**Objectives:**

We sought to characterize beverage intake among Navajo children aged 2–5 y and identify factors associated with healthier beverage habits.

**Methods:**

This observational, cross-sectional study took place from 2022 to 2023. We enrolled 80 Navajo children aged 2–5 y attending early child education programs located on Navajo Nation. Children’s primary caregivers and site staff were also enrolled. Cross-sectional surveys included the Beverage Intake Questionnaire for Preschool-aged Children. We used age-based national guidelines to determine the proportion of children meeting recommendations for sugar-sweetened beverage (SSB) intake and adequate beverage hydration.

**Results:**

In this cohort of 80 children, 10.0% met recommendations for SSB intake and 26.3% maintained adequate beverage hydration. Of all beverage types, water was consumed the most, averaging 16.7 fluid ounces per day (standard deviation 11.7). Children also consumed a daily average of 12.9 fluid ounces of SSBs (standard deviation 17.8). Younger and more physically active children were more likely to meet adequate beverage hydration guidelines. The majority of participating early child education sites adhered to national beverage-related recommendations.

**Conclusions:**

In this cohort of young Navajo children, water was consumed more than any beverage and early child education sites provided healthy beverage environments. Adequate beverage hydration, observed in 26.3% of children, was associated with younger age and greater physical activity. Establishing healthy beverage habits at an early age, ensuring access to safe drinking water, and promoting culture and tradition could sustain healthy beverage choices among American Indian children.

## Introduction

Diet-related diseases are on the rise among youth in the United States, particularly in minoritized communities affected by food and water insecurity [1,2]. Communities of color disproportionately face a combination of water insecurity, water safety concerns, and targeted marketing of sugar-sweetened beverages (SSBs) [3,4]. For this reason, child beverage consumption patterns are complex behaviors shaped by broad structural factors which, in turn, contribute to health disparities such as childhood obesity and type 2 diabetes risk [[5], [6], [7]].

Consensus guidelines state that children under 6 y should not consume any SSBs, defined as any drink with added sugar including soda, fruit drinks, sports and energy drinks, sweetened dairy drinks, and sweet teas [8]. Yet, SSBs are a leading source of energy intake, comprising 6%–15% of total caloric intake among children in the United States [9,10], with 57.1% of all children in the United States drinking SSBs daily as of 2021 [11]. Although SSB consumption among children has declined over the past several decades (from an average of 224.6 calories/d in 2003 to 132.5 calories/d in 2014) [12], a closer look actually reveals growing disparity gaps. Among 2- to 5-y-old children, Dai et al. [13] found that significant declines in SSB consumption were only observed among non-Hispanic White children from 2003 to 2018, in contrast to non-Hispanic Black and Hispanic children [13]. Similarly, among Supplemental Nutrition Assistance Program (SNAP) recipients, SSB consumption has remained unchanged from 2015 to 2019 [14]. It is also notable that the types of SSBs consumed by children have shifted over time: although soda intake has declined, fruit drink and sports drink consumption has risen in part due to changing marketing tactics toward children and their caregivers [15,16].

In addition to eliminating SSBs, recommendations for healthy beverage habits also include adequate consumption of healthy beverages to maintain hydration. Inadequate hydration can impair children’s cognitive function and school performance and negatively impact mental and physical health [17]. Although total fluid intake also takes into account water consumed in foods, consensus guidelines state that 2- to 3-y-olds should drink 8–32 fluid ounces of water daily, and 4- to 5-y-old children should drink 12–40 fluid ounces daily [8]. The upper range of these recommendations reflects the total volume consumed through water and milk combined; for example, if a 3-y-old drank 8 fluid ounces of milk on a given day, then 24 fluid ounces of water would be recommended to meet her total “healthy beverage” intake for the day. Yet, based on National Health and Nutrition Examination Survey (NHANES) data from 2009 to 2012, 54.5% of United States children aged 6–19 y are inadequately hydrated (compared with 29.5% among adults) [18]. Among younger children, the Centers for Disease Control has reported that children aged 2–5 y consume an average of 13 ± 0.6 fluid ounces of water per day, based on 2015–2018 NHANES data [19]. In both reports, lower intake is more frequent among people of color.

Despite robust findings that diet-related health conditions and their upstream determinants are disproportionately experienced by people of color in the United States, the aforementioned studies examining child beverage patterns by race/ethnicity lack American Indian and Alaska Native [20] representation [13,18,19,21]. This lack of beverage consumption data among AIAN children limits our understanding of how beverage habits may contribute to diet-related health conditions, which are disproportionately experienced among AIAN children across diverse tribal communities [2,22]. Two studies (one from rural Oklahoma in 1997, another from rural Alaska from 2005 to 2009) have described comparable SSB intake among AIAN versus White children [23,24]. A few other studies have reported noncomparative descriptions of beverage intake in small cohorts of AIAN children. Among AIAN children in Wisconsin from 2008 to 2009, LaRowe et al. [25] reported an average daily SSB consumption of 6.24 and 6.88 fluid ounces among 2- to 3- and 4- to 5-y-old children, respectively, whereas a mixed-methods study by Elwan et al. [26] found that 29% of children aged 3–11 y in rural Alaska drank SSBs (primarily juice drinks), with a mean serving size of 8.55 ± 2.91 fluid ounces. In the latter study, 19% of children did not drink water daily, likely related to caregiver concerns regarding tap water safety [26,27].

Specifically in Navajo Nation, youth are at heightened risk of developing type 2 diabetes [28], and ≤35% of families with young children may face water insecurity [29]. Yet, efforts to promote healthy beverages have been unfolding in Navajo Nation, including policy and community-based initiatives [30]; these efforts have used strength-based approaches to increase access and/or elevate the importance of water in Diné culture. For instance, Navajo Nation exercised its tribal sovereignty to pass the Healthy Diné Nation Act in 2014, placing an added tax on SSB while eliminating taxation on water, and directing revenue toward community-led wellness projects, including 14% of the tax revenue toward food and water initiatives [31]. Understanding Navajo children’s beverage habits is all the more relevant in the context of mounting efforts to promote healthy beverages. Given the paucity of data on beverage consumption patterns, our team assessed beverage consumption among children enrolled in early child education centers in Navajo Nation from 2022 to 2023. We sought to understand the proportion of children meeting dietary recommendations on beverage consumption, patterns and sources of beverage consumption, and factors associated with healthier beverage habits.

## Methods

### Study design

This observational cross-sectional study utilized baseline data collected for a prospective pilot intervention, Water is K’é, involved families with children aged 2–5 y, enrolled in 7 early child education programs during 2022 and 2023.

Our study used culturally grounded, community-based participatory approaches to inform study activities, program improvements, and advocacy for policy change. K’é served as the foundation for our asset-based approach [32]. The Diné Policy Institute defines K’é as the ancient system of kinship observed between Diné people and all living things in existence. Water is K’é elevates the central role of water to each Diné person’s identity, physical, and spiritual health. Our community-based participatory approach has embraced K’é in how we view community partners as our relatives: together we are collectively committed to make it possible for every family to raise Diné healthy children. A Community Advisory Group comprised teachers, public health professionals, caregivers, elders, and traditional knowledge holders met regularly throughout this study to provide guidance and feedback at all stages.

### Study setting

Among the estimated 174,000 individuals residing on the rural 27,000 square-mile Navajo Nation, the median household income is $27,389 with an average household size of 3.1 individuals [33]. The study setting included 7 Family and Child Education (FACE) Programs in Northern and Eastern Navajo Agencies. FACE Program is a national tribal program that provides early childhood, parenting, and adult education services. Initiated in 1990 by the Bureau of Indian Education, FACE supports families with children from prenatal to 5 y of age with both center-based and home-based programs [34]. Although FACE programs do not have an income requirement for eligibility, participants must be AIAN.

Navajo Nation has a long history of water contamination, including decades of uranium mining with unsafe levels of uranium and arsenic documented in water sources, combined with extractive mining industries, diversion and contamination of waterways, and underinvestment in water infrastructure [35,36]. Reassuringly, a national study in 2015 by the National Resources Defense Council found that Navajo Nation utilities supplied an acceptable quality of water to residents on the public water supply [37]. Nonetheless, our community needs assessment in 2016–2019 revealed that residents had continued concerns about tap water quality, resulting in greater reliance on bottled water and SSBs. Furthermore, 30% of homes on Navajo Nation do not have complete plumbing and 40% of households rely on hauled drinking water (from sources such as water-filling stations located anywhere from 10 min to an hour away) to meet their daily needs. The Office of Environmental Policy and Compliance estimates that Navajo households may spend ≤$291 per week on water [35]. Water is therefore difficult to access and costly for many families.

### Study population

From October 2021 through February 2023, we enrolled children, primary caregivers, and staff at 7 participating early child education sites. All families enrolled in participating sites were invited to take part in the study. Primary caregivers were self-identified as the adult living with and assuming primary responsibility for caring for the child. Inclusion criteria were enrollment in a participating early child education site, a child between 2 and 5 y old at enrollment, and family plans to remain at the early child education site throughout the school year.

### Data collection

Caregiver surveys included data on the caregiver (e.g., age, gender, relation to child, caregiver perceptions toward water safety, and Diné traditions); child (age, gender); and household (e.g., household size, food insecurity [38], utilization of food assistance programs). Caregivers were also asked to report on child health behaviors including beverage intake using the Beverage Intake Questionnaire for Preschool-aged Children, BEVQ-PS [39], physical activity, screen time, and sleep. We chose the BEVQ-PS questionnaire for measuring our primary outcomes based on our review of available instruments for this age group [40]. The BEVQ-PS has been validated in English, with good comprehensibility among caregivers. The questionnaire asks how often, how much (in fluid ounces), and where children are typically offered each of the following beverages: water, 100% juice, regular milk, unsweetened tea, diet soda, fruit drinks, regular soda, sweetened tea, flavored milk, sports drinks, and other (if marked, respondent is asked to describe).

Early child education staff completed an Early Child Education Site survey, which collected data on site practices (frequency and modalities for offering water to children) and environment (water and SSB sources and availability, presence of promotional materials related to beverages, and beverage-related policies at the facility). Because sites had switched from in-person to virtual learning due to COVID in March 2020 and were barely returning to in-person learning, in March 2021 teachers were instructed to respond based on typical pre-COVID activities and practices that they planned to resume upon re-opening. If multiple surveys were completed for a site, we used the survey completed by the teacher or education technician with the longest time working at the site.

Caregivers and teachers completed respective surveys using paper forms and received a $25 gift card in appreciation of their time. Data were double-entered into a secure Health Insurance Portability and Accountability Act (HIPAA)-compliant database (REDCap 13.1.29) with quality checks for entry errors, outliers, and missing data.

### Description of variables

We calculated children’s average daily water consumption and SSB consumption (fluid ounces per day), average energy intake from SSB (kilocalories per day), and average fluid ounces from water and plain milk combined. The following drinks were categorized as SSBs: fruit drinks, regular soda, sweetened tea, flavored milk, and sports drink. “Other” beverages were reviewed and categorized as follows: fruit smoothies as 100% juice, Pedialyte as a sports drink, Navajo tea as unsweetened tea, and soy and almond milk grouped with unflavored milk. SSB calculations were assigned as missing if more than 2 of the 5 SSB beverages were missing. Average energy intake for each drink was assigned based on unpublished scoring instructions provided by the authors of BEVQ [41].

We derived categorical variables for beverage and health behaviors based on national guidelines using age-based recommendations, as summarized in Table 1 [8,42]. Adequate beverage hydration was only calculated if neither water nor plain milk data were missing.

**TABLE 1:** Criteria for health behavior recommendations (average daily measures), by age.

	2- to 3-Y olds	4- to 5-Y olds
SSB consumption	None	None
Water consumption (minimum)	8 Fluid ounces	12 Fluid ounces
Adequate beverage hydration	32 Fluid ounces water/plain milk	40 Fluid ounces water/plain milk
Physical activity (minimum)	1 h	1 h
Screen time (maximum)	1 h	1 h
Sleep time (minimum)	11 h	10 h

Co-variates included characteristics of the caregiver, household, and child. Households were considered multigenerational if they included 2 or more adult generations (e.g., ≥1 adult of age 18–50 y and 1 adult >50 y) or had a “skipped” generation (i.e., primary caregiver was a grandparent).

Site survey data were used to calculate the proportion of sites meeting recommendations of the Centers for Disease Control and Prevention and Child and Adult Care Food Program guidelines to increase access to drinking water and other healthier beverages in early care and education settings [43,44] as follows:

• *Frequency of juice offerings*: Juice is limited to 4–6 oz per day for 1- to6-y-old children
• *Type of milk offered*: Toddlers 2 y of age and older are offered 1% or skim milk, or equivalent nondairy milk, at meals
• *Water availability throughout the day*: Drinking water should be readily available to children throughout the day
• *Water availability at mealtime*: Drinking water must be made available to children during mealtime; staff are not required to have water available for children to self-serve
• *Offering water to children*: Offering water means asking the children whether they would like water at different times throughout the day

### Statistical analysis

Following established scoring protocols [45], we calculated daily consumption in fluid ounces by multiplying frequency with volume, and estimated energy intake by multiplying fluid ounces by average kcals/fluid ounces per beverage type. Daily intake was not calculated if either frequency or amount was missing.

We generated descriptive output on beverage consumption patterns (types, daily intake); beverage sources, child health behaviors (average hours of physical activity, screen time, and sleep); caregiver attitudes (average Likert score on water safety perception and importance of Diné water teachings), as well as early child education site characteristics (number of times water offered per day, number of water sources, SSB sources, and beverage promotional materials at early child education sites).

Finally, we used logistic regression to assess for factors associated with adequate beverage hydration. We conducted a backward elimination analysis to identify factors associated with healthier beverage habits (adequate hydration). Starting with a logistic regression model that included all variables included in univariate analysis, we applied a significance level of 0.3 to retain variables in the model. The analyses were conducted using SAS 9.4 (SAS Institute).

### Ethical considerations and author positionality

This study was conducted according to the guidelines laid down in the Declaration of Helsinki, and all procedures involving research study participants were approved by the Navajo Human Research Review Board (NNR-21.409) and Massachusetts General Brigham Institutional Review Board (Protocol 2020P001537). Written informed consent was obtained from all subjects; for children, written informed consent was obtain from the primary caregiver.

Among the authors, 8 individuals are members of the Navajo Nation, and 6 individuals are not from Navajo. Nine of the authors live and work on or in close proximity to the Navajo Nation. In terms of study participant interactions, one Navajo study member was responsible for study enrollment, and data collection was carried out by 5 individuals (4 of whom are Navajo).

## Results

A total of 80 children were enrolled, for whom caregivers completed baseline surveys. As shown in Table 2, 51.3% of the children enrolled were 2- to 3-y old, and 53.8% were girls. Most primary caregivers were the parent of the child (86.3%) and 92.4% identified as females. Households comprised a median of 5 people, and 37.5% were multigenerational. Most families participated in the Supplemental Nutrition Assistance Program (SNAP) (57.1%), and 32.5% took part in Women Infant Children (WIC) program. Ninety percent of households had tap water in their homes, and 60.0% reported food security. Fewer than half of the respondents (42.5%) felt that their tap water was safe to drink.

**TABLE 2:** Baseline characteristics of participating families, *n* = 80.

Characteristic, *n* if other than 80	*N* (%)	Median (IQR)
Child age-		
2- to 3-y-old	41 (51.3)	
4- to 5-y-old	39 (48.8)	
Child gender		
Male	37 (46.2)	
Female	43 (53.8)	
Health habits		
Hours of physical activity (*n* = 78)		1 (0.5–1)
Hours of sleep (*n* = 69)		9 (8–11)
Hours of screen time (*n* = 79)		2 (1–3)
Caregiver age (*n* = 77)		
All caregivers		30 (27–35)
Parent		29.5 (27–34)
Other		46 (29–55)
Caregiver gender (*n* = 79)		
Male	6 (7.6)	
Female	73 (92.4)	
Caregiver’s relationship to child		
Parent	69 (86.3)	
Grandparent	6 (7.5)	
Other	5 (6.3)	
Caregiver’s highest education		
Did not finish high school	11 (13.8)	
High school graduate or GED	14 (17.5)	
Some college	38 (47.5)	
Technical or vocational degree	10 (12.5)	
Bachelor’s degree or higher	7 (8.8)	
Household size (*n* = 77)		5 (4–6)
Multigenerational household	30 (37.5)	
Number of members by age (y) per household (*n* = 76)		
Children < 6	76 (100)	1 (1–2)
Children 6–17	39 (51.3)	1 (1–2)
Adults 18–49	73 (96.1)	2 (2–2)
Adults 50+	31 (40.8)	2 (1–2)
Take part in ≥1 Food Assistance Program or Benefit	52 (65.0)	
Food assistance programs and benefits (*n* = 77)		
Food stamps (SNAP)	44 (57.1)	
Commodities (Food Distribution Program on Indian Reservations)	0 (0)	
Women Infant and Children Program	25 (32.5)	
Cash assistance/temporary assistance for needy families	8 (10.4)	
Other	2 (2.6)	
No benefits	25 (32.5)	
Piped/plumbed/tap water in home	72 (90.0)	
Believe their tap water is safe to drink	34 (42.5)	
Knowledgeable about Diné traditions about water	47 (58.8)	
Diné water traditions influence drinks they offer their child	41 (51.2)	
Food security status		
Food secure	48 (60.0)	
Food insecure	28 (35.0)	
Very food insecure	4 (5.0)	

Abbreviation: SNAP, Supplemental Nutrition Assistance Program; GED, General Education Development.

**FIGURE 1:**
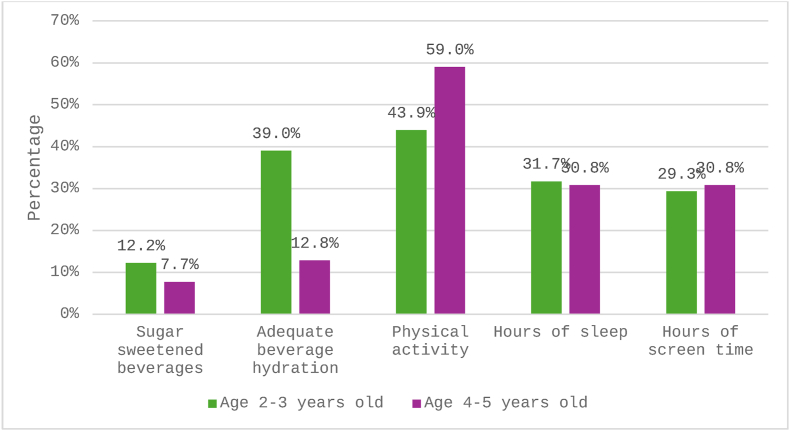
Percent of 2- to 5-y-old children meeting recommended guidelines, *n* = 80.

Consensus guidelines recommend that children avoid SSB intake and maintain adequate beverage hydration (≥32–40 fluid ounces of combined water and milk). Among all children aged 2–5 y, 10.0% met recommendations for SSB intake and 26.3% maintained adequate beverage hydration. As shown in Figure 1, fewer older children (aged 4–5 y) met these recommendations compared with younger children (aged 2–3 y). For other health habits, 43.9% of 2- to 3-y olds and 59% of 4- to 5-y olds met recommendations for physical activity, 31.7% of 2- to 3-y olds and 30.8% of 4- to 5-y olds met recommendations for sleep, and 29.3% of 2- to 3-y olds and 30.8% of 4- to 5-y olds for recommended screen time.

**FIGURE 2:**
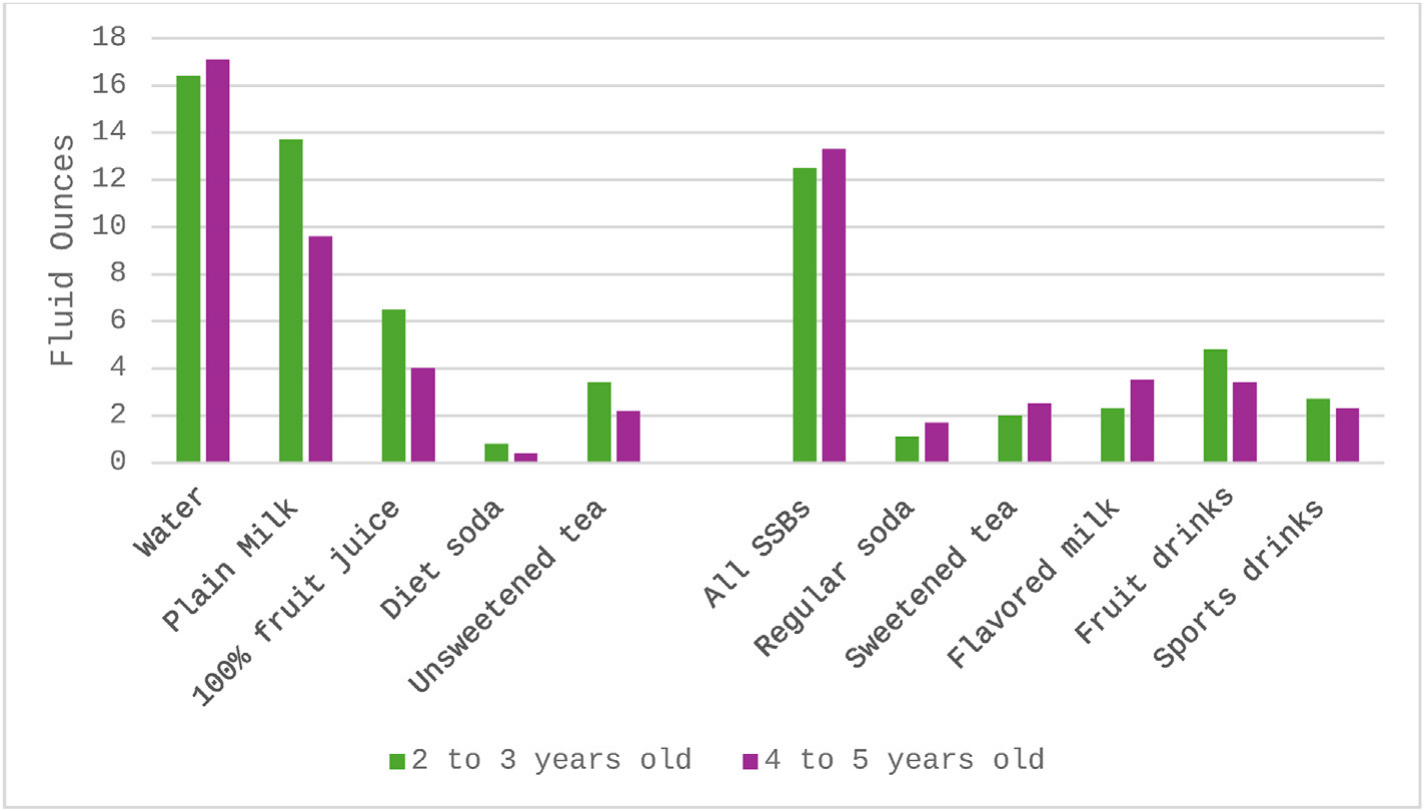
Average daily beverage intake (fluid ounces) among children by age group, *n* = 80. SSBs, sugar-sweetened beverages.

Figure 2 and Supplemental Table 1 summarize the average daily intake by beverage type. Among all beverage types, water was consumed the most with an average daily intake of 16.7 fluid ounces with a standard deviation (STD) of 11.7, and similar intake in both age groups. Plain milk was the second most consumed beverage, with an average daily intake of 11.7 fluid ounces (STD 11.3), with younger children drinking more milk than older children (13.7 vs. 9.6 fluid ounces). Although healthier beverages (water, milk, 100% juice) were consumed most, children also consumed a daily average of 12.9 fluid ounces (STD 17.8) of all SSBs combined. The average energy intake from SSB was 174.3 calories (STD 244.9).

**FIGURE 3:**
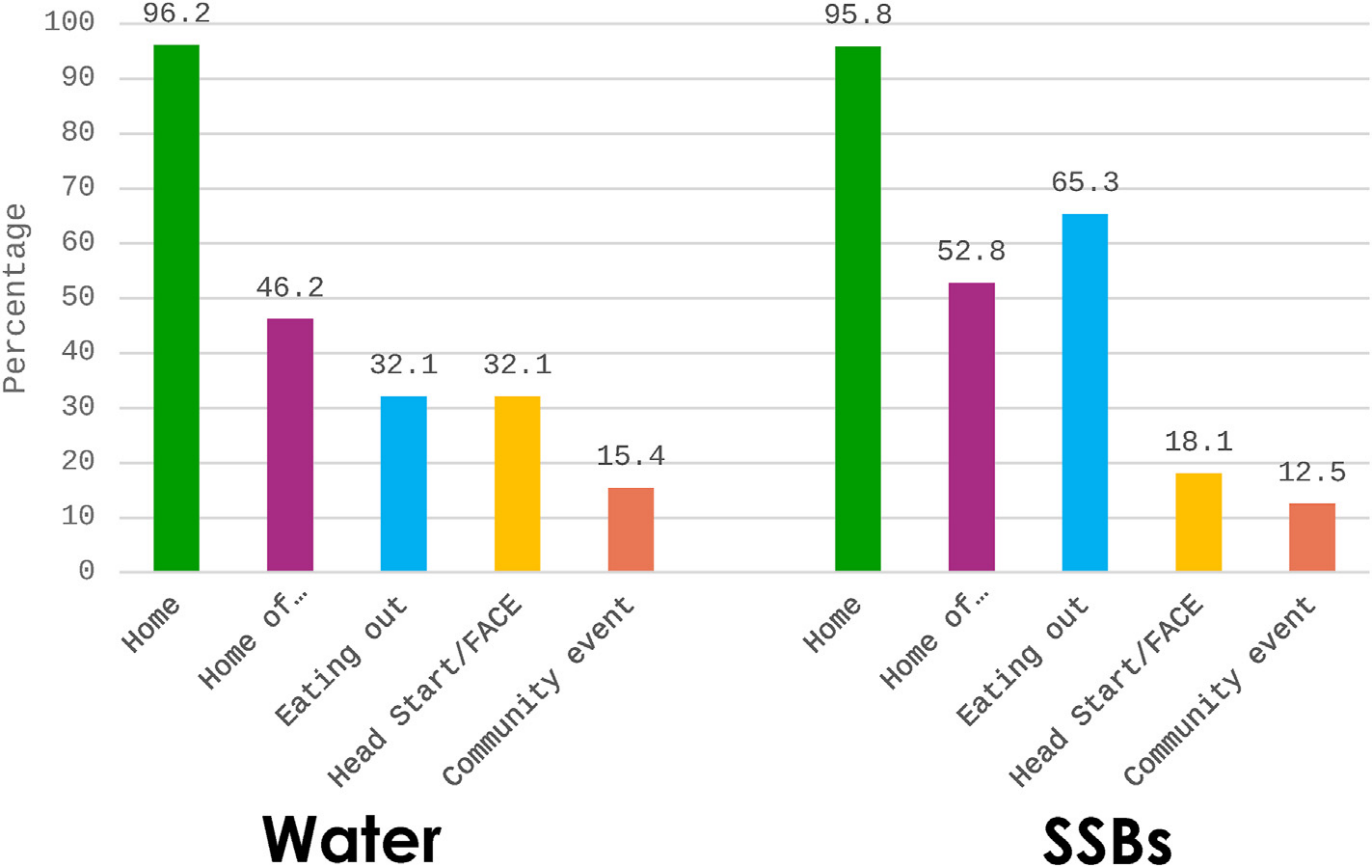
Settings where water and SSBs are offered to children (can be multiple), *n* = 80. FACE, family and child education; SSBs, sugar-sweetened beverages.

Figure 3 represents the settings in which children were typically offered water and SSBs. Both water and SSBs were available in most homes. In other settings, such as with friends and family or when eating out, children were offered SSBs more frequently than water. However, water was offered more at early child education sites and community events.

As shown in Table 3, younger children (aged 2–3 vs. 4–5 y) were more likely to meet adequate beverage hydration guidelines, with an adjusted odds ratio (OR) of 6.8 and 95% confidence interval (CI) of 1.6–28.2. In addition, children who met recommendations for physical activity were more likely to adequately hydrate (adjusted OR, 5.8; 95% CI: 1.3, 25.2). Adequate beverage hydration was also more likely in families with multigenerational households and influence of Diné traditions on beverage offerings to the child (all with odds ratios ≥ 2.0), but these were not statistically significant.

**TABLE 3:** Factors associated with adequate beverage hydration among 2- to 5-y-old children, unadjusted and adjusted models, *n* = 74.

	Adequate hydration *N* (%) *N* = 21	Inadequate hydration *N* (%) *N* = 53	Unadjusted odds ratio (95% CI)	Adjusted odds ratio (95% CI)
Younger child (aged 2–3 vs. 4–5 y)	16 (76.2)	23 (43.4)	4.2 (1.3, 13.1)^1^	6.8 (1.6, 28.2)^1^
Female child gender	12 (57.1)	31 (58.5)	0.9 (0.3, 2.6)	
Younger caregiver (under 30 vs. 30+ y)	11 (52.4)	21 (39.6)	2.0 (0.7, 5.9)	
Female primary caregiver	19 (90.5)	48 (90.6)	0.8 (0.1, 4.7)	
Caregiver with higher education^2^	15 (71.4)	38 (71.7)	1.0 (0.3, 3.0)	0.3 (0.1, 1.6)
Smaller household size (under 5 vs. 5+ y)	7 (33.3)	19 (35.8)	1.0 (0.3, 3.0)	2.3 (0.5, 9.5)
Multigenerational household	10 (47.6)	16 (30.2)	2.1 (0.7, 5.9)	3.8 (0.9, 16.5)
Enrolled in SNAP	10 (47.6)	30 (56.6)	0.7 (0.3, 1.9)	
Enrolled in WIC	9 (42.9)	15 (28.3)	1.9 (0.7, 5.4)	
Household food security (vs. food insecure)	12 (57.1)	34 (64.2)	0.7 (0.3, 2.1)	
Meets recommendations for physical activity	15 (71.4)	24 (45.3)	3.0 (1.0**,** 9.0)^1^	5.8 (1.3**,** 25.2)^1^
Meets recommendations for sleep	5 (23.8)	19 (35.8)	0.6 (0.2, 1.8)	
Meets recommendations for screen time	6 (28.6)	14 (26.4)	1.1 (0.4, 3.4)	
Piped/plumbed/tap water in home	20 (95.2)	47 (88.7)	2.1 (0.2, 19.4)	
Caregiver feels their tap water is safe to drink	11 (52.4)	22 (41.5)	1.5 (0.6, 4.3)	
Caregiver knowledgeable about Diné traditions	12 (57.1)	31 (58.5)	0.9 (0.3, 2.6)	
Diné traditions influence drinks offered to child	14 (66.7)	24 (45.3)	2.4 (0.8, 6.9)	2.7 (0.7, 9.7)

SNAP, Supplemental Nutrition Assistance Program; WIC, women, infants, and children.

1 Values are significant.

2 Higher education includes some college, technical or vocational school, or higher degree.

The majority of FACE Programs on Navajo Nation adhered to beverage-related recommendations (Supplemental Table 2) in terms of frequency of fruit juice offerings (100%), type of milk offered (86%), water availability throughout the day (86%), water availability at mealtime (57%), and offering water to children (100%). In terms of beverage resources and policies, none of the facilities had soda or other vending machines at entrances or other public areas, and only one had a machine on site. Two-thirds of programs had a beverage policy (67%) in place.

## Discussion

Our cross-sectional study characterized beverage habits among 80 children enrolled in Navajo FACE programs. Of all beverage types, water was consumed the most, with an average intake of 16.7 fluid ounces per day (or 2.1 8-ounce servings). Although the amount of water consumed was similar across age groups, other beverage consumption patterns differed with age, with younger children drinking more plain milk, 100% fruit juice, and fewer SSBs. As a rough comparison, using NHANES data of same-age children in the United States from 2009 to 2014 (which uses 24-h dietary recall rather than BEVQ-PS’s past-month recall), Navajo children drank more water on average (16.4 vs. 11.2 fluid ounces) and more SSBs (12.5 vs. 4.8 fluid ounces) [46].

Adequate hydration is associated with improved cognition and school performance among children [17]. We found that 39.0% and 12.8% of 2- to 3-y olds and 4- to 5-y olds, respectively, achieved adequate beverage hydration. These findings reinforce the public health significance of inadequate hydration among children. Based on urine osmolality, 54.5% of children aged 6–19 y in the United States are inadequately hydrated, with higher rates among children from communities of color [18]. In our cohort, younger children and more physically active children were more likely to meet adequate beverage hydration recommendations. Although not significant, adequate beverage hydration was also associated with multigenerational households and caregivers who were influenced by Diné traditions. These findings suggest that stronger intergenerational support and connections to indigenous culture may be protective factors for early child health, consistent with other findings in the literature. Although not unique to indigenous cultures, the central family role of indigenous elders in transmitting culture, language, and values is well described [47,48]. Similarly, cultural connectedness is protective across a broad range of health outcomes among indigenous children [[49], [50], [51], [52]].

The average daily intake of SSBs was 12.5 fluid ounces (STD 18.6) among 2- to 3-y olds and 13.3 fluid ounces (STD 17.3) among 4- to 5-y olds. Only 10.0% of all children reported no SSB consumption (meeting American Academy of Pediatrics recommendations). Children were offered SSBs at home and often when eating out, but rarely at Early Child Education sites or community events. Involving caregivers and household family members can help to create a healthy home environment, engage children, and sustain their healthy behaviors [53,54]. Based on national NHANES data, 61.5% of 2- to 5-y olds and 72.1% of 6- to 11-y olds reported daily SSB consumption, with this percentage rising to 76.3% among 12- to 19-y olds [55]. Given evidence that SSB intake increases as children get older, intervening early may have the greatest impact on lifelong healthy beverage habits [56].

FACE programs operating on Navajo Nation generally provided healthy beverage environments, particularly in terms of the types of beverages offered and availability and offering of water to children. Most sites relied on bottled water as the primary source of drinking water, reflecting a practical solution to staff and caregiver concerns regarding tap water safety. Given only 17% of staff felt knowledgeable about Diné traditions about water and 43% of sites had limited educational materials, opportunities to support Early Child Education sites include providing additional educational materials to promote healthy beverages as well as staff training or lesson plans related to Diné tradition and culture.

Our study has both strengths and limitations. We successfully used a validated questionnaire (BEVQ-PS). To help with administration, we included pictures of each beverage type and demonstrations of beverage amounts. Although somewhat complicated to administer, complete response rates were high, which allowed us to calculate average daily water and SSB intake in 94% of respondents. However, surveys were administered cross-sectionally during the fall/winter season and therefore do not reflect seasonal variations. Furthermore, although the BEVQ-PS is a validated instrument, results rely on caregiver self-report; for children who spend time away from their primary caregiver (for instance, attending an early child education center), caregivers may have only partial knowledge of their child’s beverage intake. However, given the results of the Early Child Education survey, we do not suspect that children were exposed to excessive SSBs in the Early Child Education setting. The small sample size limited our ability to confirm whether several factors were significantly associated with healthy beverage habits, e.g., multigenerational households and the influence of Diné traditions. Finally, given this cohort reflects a small number of children enrolled in FACE programs, our findings may not be representative of all Navajo children in this age group.

In conclusion, in this cohort of young Navajo children, water was consumed more than any other beverage type. Intervening at an early age (0–4) could establish healthy habits, including adequate beverage hydration. Ensuring access to safe and clean drinking water and promoting culture and tradition will be key in helping indigenous communities sustain healthy choices in children and communities. Future research should explore interventions to engage children and their caregivers to promote lifelong healthy beverage habits.

## Author contributions

The authors’ responsibilities were as follows – CVG, CH, KH, LV, SSS, KB, EB, LB, ME, and BJ: designed research; CVG, SSS, BJ, RW, ASY, and L Tsosie: conducted research; LV, L Tsosie, L Trevisi, BJ, and SSS analyzed data; and SSS, LTrevisi, and BJ: wrote the article; SSS and CVG: had primary responsibility for the final content; and all authors read and approved the final manuscript.

## Funding

This research was supported by grant #77234 from Healthy Eating Research, a national program of the Robert Wood Johnson Foundation; Robert Wood Johnson Foundation had no role in the design; collection, analysis, and interpretation of data; writing of this article, nor restrictions regarding submission of the report for publication.

## Data availability

All data from research conducted on the Navajo Nation belongs to the Navajo Nation. Data described in the manuscript, code book, and analytic code will be made available upon request, pending approval from the Navajo Nation Human Research Review Board.

## Ethical standards disclosure

This study was conducted according to the guidelines laid down in the Declaration of Helsinki and all procedures involving research study participants were approved by the Navajo Human Research Review Board (NNR-21.409) and Massachusetts General Brigham Institutional Review Board (Protocol 2020P001537). Written informed consent was obtained from all subjects/patients; for children, written informed consent was obtained from the primary caregiver.

## Declaration of Generative AI in Scientific Writing

Authors did not use AI and AI-assisted technologies in the writing process.

## Conflict of interest

The authors report no conflicts of interest.
