# Supplementary material for: Beverage Consumption Patterns Among Navajo Children Aged 2–5 Years
Source: Curr Dev Nutr. 2024 Oct 26;9(3):104493. doi: 10.1016/j.cdnut.2024.104493 (PMC11938074; doi:10.1016/j.cdnut.2024.104493)
Supplement: multimedia component 1 [file mmc1.docx]

**Supplemental Table 1. Average daily beverage intake in fluid ounces among children ages 2 to 5 years old, n=80**

|  | **2 to 3 years old** | **4 to 5 years old** | **All children** |
| --- | --- | --- | --- |
| **Daily intake (fluid ounces)** | **Mean (STD)** | **Mean (STD)** | **Mean (STD)** |
| Water (n=76) | 16.4 (10.5) | 17.1 (13) | 16.7 (11.7) |
| Plain Milk (n=76) | 13.7 (11.5) | 9.6 (10.8) | 11.7 (11.3) |
| 100% fruit juice (n=75) | 6.5 (8.2) | 4 (4.8) | 5.2 (6.8) |
| Diet soda (n=79) | 0.8 (3.6) | 0.4 (1.4) | 0.6 (2.8) |
| Unsweetened tea (n=75) | 3.4 (8.2) | 2.2 (7.3) | 2.8 (7.7) |
| All SSBs (combined) (n=75)* | 12.5 (18.6) | 13.3 (17.3) | 12.9 (17.8) |
| Regular soda (n=75) | 1.1 (3.4) | 1.7 (4.1) | 1.4 (3.7) |
| Sweetened tea (n=74) | 2 (6.1) | 2.5 (5.3) | 2.3 (5.6) |
| Flavored milk (n=74) | 2.3 (6.3) | 3.5 (8.3) | 2.9 (7.3) |
| Fruit drinks (n=74) | 4.8 (8.9) | 3.4 (5) | 4.1 (7.2) |
| Sports drinks (n=73) | 2.7 (6.4) | 2.3 (4.5) | 2.5 (5.5) |
| **Average energy intake (calories)** |  |  |  |
| All SSBs (combined) (n=75)* | 170.1 (259.9) | 178.6 (232) | 174.3 (244.9) |

**Supplemental Table 2. Early Child Education Site Practices, n=7**

| Response (n if not 7) | **All sites** |
| --- | --- |
| **Frequency of beverage offerings** | |
| **100% fruit juice** | **Meet Recs: 100%** |
| 2 times per day or more | 0 (0%) |
| 1 time per day | 0 (0%) |
| 3 -4 times per week | 4 (57%) |
| 2 times per week or less | 3 (43%) |
| **Sugary drink** |  |
| 1 time per month or more | 0 (0%) |
| 1 time every few months | 1 (14%) |
| 1-2 times per year | 3 (43%) |
| Never | 3 (43%) |
| **Type of milk offered to children 2 years and older** | **Meet Recs: 6 (86%)** |
| Whole (regular) | 2 (29%) |
| Reduced fat | 4 (57%) |
| Low fat | 5 (71%) |
| Fat free / skim | 1 (14%) |
| **Flavored milk** |  |
| 1 time per day or more | 1 (14%) |
| 3-4 times per week | 1 (14%) |
| 1-2 times per week | 4 (57%) |
| Never | 1 (14%) |
| **Water, n=6** |  |
| 4 times per day or more | 5 (83%) |
| 3 times per day | 1 (17%) |
| 2 times per day | 0 (0%) |
| 1 time per day or less | 0 (0%) |
| **Water Promotion and Access** | |
| **Main source of drinking water** |  |
| Unfiltered tap water | 1 (14%) |
| Filtered tap water | 2 (29%) |
| Bottled water | 4 (57%) |
| Other (specify) | 0 (0%) |
| **Drinking water is available** | **Meet recs: 6 (86%)** |
| Only when children ask | 0 (0%) |
| Only when children ask and during water breaks | 1 (14%) |
| Only indoors, always visible and freely available | 3 (43%) |
| Indoors & outdoors, always visible / freely available | 3 (43%) |
| **Drinking water availability with meals or snacks** | **Meet Recs: 5 (57%)** |
| Not provided at the table at meals or snacks | 2 (29%) |
| Provided at the table with meals | 2 (29%) |
| Provided at the table with snacks | 0 (0%) |
| Provided only after child finishes milk or juice | 0 (0%) |
| Provided only after child finishes meal or snack | 0 (0%) |
| Children allowed only one serving | 0 (0%) |
| Children allowed to self-serve as much as want | 5 (71%) |
| Provided only upon request by child | 0 (0%) |
| **During day, teachers remind children to drink water, n=6** | **Meet recs: 100%** |
| Rarely or never | 0 (0%) |
| Sometimes | 1 (17%) |
| Often | 5 (83%) |
| **Beverage Resources and Policies** | |
| **Posters, books, other learning materials promote healthy beverage habits** |  |
| Few or no materials | 2 (29%) |
| Some materials with limited variety | 2 (29%) |
| A variety of materials | 3 (43%) |
| Large variety of materials, new items added / rotated seasonally | 0 (0%) |
| **Soda and other vending machines are located** |  |
| In the entrance or front of building | 0 (0%) |
| In public areas, but not entrances | 0 (0%) |
| Out of sight of children and families | 1 (14%) |
| No vending machines on site | 6 (86%) |
| **Staff feels that tap water is safe to drink, n=6** |  |
| Agree | 3 (50%) |
| Neither agree nor disagree | 3 (50%) |
| Disagree | 0 (0%) |
| **Staff feels knowledgeable about Diné traditions about water, n=6** |  |
| Agree | 1 (17%) |
| Neither agree nor disagree | 3 (50%) |
| Disagree | 2 (33%) |
| **Staff believes Diné traditions influence what drinks they choose to offer their children, n=6** |  |
| Agree | 3 (50%) |
| Neither agree nor disagree | 3 (50%) |
| Disagree | 0 (0%) |
| **Early childhood education policies, n=6** |  |
| Foods provided to children | 0 (%) |
| Beverages provided to children | 2 (33%) |
| Creating healthy mealtime environments | 3 (50%) |
| Teacher practices to encourage healthy eating | 2 (33%) |
| Not using food to calm /encourage good behavior | 1 (17%) |
| Planned, informal nutrition education for children | 2 (33%) |
| Professional development on child nutrition | 1 (17%) |
| Education for families on child nutrition | 2 (33%) |
| Guidelines for foods at holidays and celebrations | 1 (17%) |
| Fundraising with non-food items | 0 (0%) |
| **Early childhood education policies # above topics included, n=6** |  |
| None or no policy | 2 (33%) |
| 1-4 topics | 3 (50%) |
| 5-8 topics | 1 (17%) |
| 9-10 topics | 0 (%) |
